# Enablers and barriers to Electronic Community Health Information System (eCHIS) usage in Kenya: A Work System Framework Analysis of frontline health worker experiences

**DOI:** 10.1371/journal.pone.0358994

**Published:** 2026-10-08

**Authors:** Michael Kagiri, Peter Wagacha, Daniel Ochieng, Salome Ireri, Michael Anindo, Maureen Adoyo, Rachael Wanjiru, Joan Kirui, Raphael Pundo, Maureen Kimani, John Wanyungu

**Affiliations:** 1 Department of Computing & Informatics, University of Nairobi, Nairobi, Kenya; 2 Department of HealthIT, University of Nairobi, Nairobi, Kenya; 3 Division of Community Health Services, Ministry of Health, Nairobi, Kenya; University of Global Health Equity, RWANDA

## Abstract

**Introduction:**

The Electronic Community Health Information System (eCHIS) is Kenya’s flagship digital health solution rolled out to strengthen community health service delivery and accelerate progress towards achieving universal health coverage. Despite its widespread rollout, evidence from global health initiatives affirms that the mere presence of a system doesn’t guarantee meaningful use. Consistent eCHIS usage by frontline health workers remains a critical determinant of realising anticipated benefits, underscoring the need to understand the context-dependent enablers and barriers influencing such consistent usage, based on users’ lived experiences.

**Methods:**

The study employed a qualitative phenomenological design in three counties, conducting 22 interviews. A total of 12 Community Health Promoters (CHPs), 6 Community Health Assistants (CHAs), and 4 county focal persons participated in the study. The qualitative data were transcribed and analysed thematically using NVivo, guided by the Work System Framework (WSF), which examined usage across four dimensions: participants, information, technology, and processes.

**Results:**

The findings showed that sustained eCHIS usage is influenced by a set of interrelated socio-technical enablers and barriers across the WSF dimensions. The key enablers included incentives, supportive supervision, training opportunities, real-time data access, improved accuracy, device portability, transparency, and data ownership. The major barriers included inadequate and unequal training, household resistance to disclosing sensitive information, financial constraints, device failures, data synchronisation issues, missed household visits due to environmental challenges, and delayed system updates.

**Conclusion:**

The study’s contribution was to show that sustained system use is shaped by interactions among work system dimensions rather than by technology availability alone. Through the WSF, the study provides a holistic understanding of the socio-technical determinants that influence the sustained use of national digital health systems. The findings affirm the need for refresher training, infrastructure support, workflow alignment, and community trust to ensure sustained use of digital health systems in low-resource settings.

## Introduction

Kenya’s health system is a devolved function structured into six levels: community, dispensary, health centre, sub-county hospital, county referral hospital, and national referral hospitals. Of the six levels, Level 1 facilities (community health units) serve approximately 5,000 people [1] and are linked to primary healthcare facilities (KEPH levels 2–4). Each of the CHUs is responsible for community-level services, is served by frontline community health promoters (CHPs), is supervised by community health assistants (CHAs), and is coordinated at the county level by community health focal persons [2]. In Kenya, the backbone of community health services began with frontline community health workers (CHWs), who were selected by their communities and trained to provide basic healthcare services, health education, and referrals to local households without any formal compensation. Despite this, many exceeded their expected two-hour commitment, often juggling health duties alongside other jobs and family commitments. The CHWs were required to manually record and input health data into large, cumbersome ministry-approved books. However, this changed in 2017, when their role evolved into that of CHPs, accompanied by a monthly stipend of Ksh 2,500(USD 19.3) by the national government and a similar amount from the county governments to support their work [3]. Starting in 2021, the government, with the assistance of Living Goods, a non-governmental organisation (NGO), provided the CHPs with smartphones that enabled them to upload household health data directly into the eCHIS.

Since that period, Kenya has intensified its efforts to provide an enabling environment for digital health integration at the community level. Within this, key initiatives, the National Community Health Digitization Strategy (2020–2025), Health Act 2017 and the Digital Health Act 2023 [4] were launched. The National Community Health Digitization Strategy aimed at providing the blueprint for the development and implementation of a standardized national eCHIS; the Digital Health Act 2023 laid the legal framework for digital health services, digital health agency, and integrated digital health information systems [5,6]. This informed the roll-out of eCHIS at Level 1 (community level), which has been critical in providing more efficient and effective healthcare delivery at the community level by supporting household registration, service delivery tracking, referrals, and reporting. It’s through such initiatives that the government implemented eCHIS, *“a digital solution developed to support quality community health service delivery in the Republic of Kenya by providing client management with decision support, community-based surveillance, commodity management, performance management, and automated data management processes and tools, messaging, and e-learning capability.”* This eCHIS holds the potential to enhance care, especially in resource-limited community settings, improve data accuracy and integrity, and enhance decision-making at the community level compared to traditional paper-based systems [7].

Digital health solutions such as eCHIS have been associated with enhanced data-driven decision-making, streamlined patient referral processes, improved patient monitoring, reduced redundancy in recording and reporting, and strengthened collaborations amongst healthcare providers [7].

In recognition of the role of information and communication technology (ICT) as a key enabler for universal health coverage (UHC) [4], the Kenyan government has invested significantly in implementing eCHIS nationwide to enhance the delivery of community health services [8]. Prior to this implementation of eCHIS, community health data collection was largely manual, often untimely, inaccurate, and incomplete, affecting decision-making and subsequent community interventions that could have otherwise been prevented earlier [9].Through its implementation, it has become a critical tool for tracking UHC progress and strengthening decision-making at the community level [10].

However, despite these investments and the potential of digital health systems, the realisation of eCHIS’s benefits depends not merely on its rollout but on its consistent and meaningful use in routine service delivery by the frontline workers. Previous research has shown that implementing digital health systems does not necessarily result in sustained use. For instance, studies on eCHIS usage in Ethiopia by Bogale [11] reported over 90% eCHIS acceptance rates, yet only half (50.1%) of the health extension workers (HEWs) consistently used eCHIS in their routine work. This divergence demonstrates that the willingness to use a digital health system does not necessarily translate into sustained use during service delivery. Further, a study by Ngugi [12] affirms that a health information system may or may not be used as intended, depending on various enablers and barriers. Thus, to better understand eCHIS usage requires understanding these determinants that enable or constrain sustained eCHIS usage by the frontline health workers. Additionally, Burton-Jones and Straub [13] argue that the system usage construct is context-dependent, necessitating a grounded, experience-based understanding of what promotes or hinders consistent eCHIS usage to ensure the consistent delivery of community healthcare services.

This gap between system availability and actual usage implies that a range of socio-technical factors beyond system availability influence consistent eCHIS usage. In this study, eCHIS usage, being represented as the consistent incorporation of eCHIS into the routine delivery of community health services, is a critical determinant of whether the benefits are attainable. The existing literature on health information systems has identified several socio-technical factors that influence consistent system use. These include user training [7,14], infrastructure [7], system usability [7], supervision [7,14] and workload [11]. This disparity suggests that while health workers appreciate the potential benefits of eCHIS, various barriers, namely inadequate infrastructure and resources, parallel documentation, both manual and electronic, supervision, low-level training, follow-up, and increased CHPs’ workload [7,15], hinder their full utilisation in their daily operations. Understanding this gap is crucial for addressing the barriers that hinder effective eCHIS utilisation, which is essential for improving community health outcomes. Moreover, technology-related challenges equally represent a significant barrier to consistent usage of eCHIS. For instance, inadequate internet connectivity, insufficient hardware, and limited technical support were identified as obstacles to the effective use of eCHIS in community settings in Ethiopia [7].

Existing studies have identified numerous factors on digital health system usage, including system functionality, user training, technical support, human factors, infrastructure, and Electronic Medical Records (EMRs) operational mode [12]. However, these factors are commonly examined in isolation and offer limited explanation of how participants, information, work processes, and technologies jointly interact in real-world settings to shape system usage. An ongoing debate exists in information systems research: some scholars view information systems as technological artefacts, while others view them as socio-technical phenomena shaped by the interaction between participants, information, technology, and work processes [16]. Evidence from studies on eCHIS usage in Ethiopia shows that even well-designed systems with high user acceptance may remain suboptimal when they aren’t well-aligned with routine work processes, receive inadequate user support, or generate unreliable information not perceived as useful for service delivery [17]. Thus, eCHIS usage is not simply a dichotomy between use/non-use but rather a deeply embedded construct within the lived realities of frontline workers, shaped by distinct socio-technical determinants.

Whereas previous studies have examined determinants of digital health systems usage, important gaps remain in understanding sustained eCHIS usage based on users’ lived experiences. Theoretically, few studies have applied an integrated socio-technical lens such as the WSF to explain enablers and barriers to eCHIS usage based on the interactions of participants, information, technology, and work processes. Analytically, existing studies have documented enablers and barriers as discrete factors, offering limited understanding of how they interact within the broader work system in which digital health systems are used. Empirically, there is little qualitative evidence explaining how frontline health workers use eCHIS after its implementation within a devolved healthcare system and a formal monthly stipend, leaving important gaps in understanding their lived experiences.

In order to capture this perspective guided by the WSF as our analytical lens, the study seeks to extend system usage research by conceptualising eCHIS usage as a socio-technical phenomenon shaped by the interaction of participants, information, technology, and work processes. Secondly, it applied the WSF to explore the enablers and barriers influencing eCHIS usage in Kenya from the perspective of those directly interacting with the system. The WSF, developed by [18], conceptualises an information system as a work system comprising participants, information, technologies, and processes that jointly interact to deliver services. In the community health context, household visits represent the community service delivery process, frontline workers represent the participants, household information represents the information, and the eCHIS platform and devices represent the technology. The process dimension is the one through which CHPs deliver services, while the other dimensions shape or constrain that process. Using qualitative data on frontline workers’ lived experiences with eCHIS across three counties, the study explores the key enablers and barriers to consistent eCHIS usage. Within it, the study explores how these determinants manifest across the four work system dimensions to influence consistent eCHIS usage based on the lived experiences of the frontline workers in the selected counties.

## Methodology

### Study design, setting and period

The research adopted a qualitative phenomenological design, which focuses on understanding the participants’ experiences with eCHIS. This approach is particularly suitable for exploring complex human interactions with technology, as it allows for an in-depth examination of the meanings participants ascribe to their experiences. We employed this technique to investigate the lived experiences of frontline health workers who use eCHIS in their daily jobs. The study was conducted in three counties (Kakamega, Kisumu, and Migori) in Kenya between March 6, 2024, and April 10, 2024, where the Electronic Community Health Information System (eCHIS) had been implemented and had been in use for more than 17 months. The counties were purposively selected because they were among the early users of the eCHIS system and had been using it for a longer period, making them suitable for studying factors influencing sustained system use rather than initial utilisation. Further, participants assigned to respective community health units (CHUs) within each county were selected based on CHUs’ linkage to the Kenya Essential Package for Health (KEPH) Level 2, Level 3, and Level 4 health facilities.

### Study population and eligibility criteria

The study participants consisted of CHPs, who are the primary users of eCHIS and actively use it in their routine community health service delivery, household registration, household visits, follow-ups, and reporting. The CHAs are tasked with the supervision of CHPs via the eCHIS dashboards. Data were gathered from frontline health workers who were willing, aged 18 or older, and provided written informed consent. Regarding the sample size, the study applied saturation, being the most common guiding principle for determining the adequacy of purposive samples in qualitative research. Additionally, most research implies that a range of interviews 9–17 is acceptable to reach saturation [19].

### Sampling technique and procedures

The study employed purposive sampling to select counties and participants. The purposive sampling technique was selected as the study aimed to capture rich, relevant insights from participants based on their lived experiences using eCHIS. Only respondents were selected from regions where eCHIS had been consistently used for at least 17 months. Thus, in this study, we conducted 22 in-depth interviews comprising CHPs (12), CHAs (6), and focal persons (4) as per Table 1.

**Table 1:** Participant characteristics.

Participant Type	Number	County
CHPs	12	Kisumu, Migori, Kakamega
CHAs	6	Kisumu, Migori, Kakamega
Focal Persons	4	Kisumu, Migori, Kakamega

### Data collection tools and procedures

An in-depth interview (IDI) guide was developed, reviewed by experts, and used in the study. The guide included questions that addressed the enablers and barriers to consistent eCHIS use. In order to undertake the above, participants were recruited between March 6, 2024, and April 10, 2024, through written invitations shared via the Chief Officer of Public Health in each participating county. On the day of data collection undertaken by trained data collectors, participants received an information sheet outlining the study’s purpose, procedures, confidentiality, and voluntary nature of participation. Further, written informed consent was obtained from all participants before the interviews, with each signing a consent form that also authorised the audio recording for accuracy. The study did not interview any minors for qualitative data collection. The interviews were subsequently recorded, and supplementary notes were taken during the interviews.

### Data analysis

The data collected from the interviews were analysed using thematic analysis, following the steps outlined by [20]. The recordings were transcribed into a textual format using the Otter tool. The data was then migrated to nVIVO software for coding and analysis. The WSF guided the thematic analysis, with data mapped to each WSF dimension (participants, technology, information, and processes). This mapping allowed us to systematically examine how each enabler and barrier influenced the eCHIS usage.

### Ethical considerations

Ethical approval was obtained from Chuka University institutional review board (ref:NACOSTI/NBC/AC-0812). Participants received their written informed consent in a confidential environment. They were informed about the study’s benefits, hazards, confidentiality measures, and the right to withdraw without justification. In order to ensure confidentiality and anonymity, the participants were assigned unique codes, i.e., Res1, Res2, Res3, and the quotes were anonymised to prevent direct or indirect identification of the participants. The study ensured that real names, facility names, or identifiable personal information were not recorded in the transcripts. Thereafter, each transcript was assigned a unique code.

## Results

### Participant characteristics

As shown in Table 2, a total of 22 key informants across Kisumu, Migori, and Kakamega counties participated in the study, representing diverse roles and operational levels within the community health system as well as capturing experiences from different eCHIS implementation contexts across those counties. Out of the 22 key informants, there were 12 Community Health Promoters (CHPs), six Community Health Assistants (CHAs), and four county-level community health focal persons. The CHPs formed the largest sample, as they are the primary frontline users of the eCHIS for household registration, household visits, service delivery, and reporting. Moreover, CHAs serve as primary supervisors, data verifiers, and technical guides for CHPs, while focal persons provide supportive supervision, performance monitoring, and system administration at the county level.

**Table 2:** Characteristics of study participants.

Category	Number (n)	Percentage (%)	Counties Represented
CHPs	12	54.5	Kisumu, Migori, Kakamega
CHAs	6	27.3	Kisumu, Migori, Kakamega
FPs	4	18.2	Kisumu, Migori, Kakamega
**Total**	**22**	**100.0**	**Three counties**

### Thematic findings

The analysis of the enablers and barriers to eCHIS usage found several significant determinants divided into four dimensions: participant, information, technology features, and process. These findings highlight the enablers of eCHIS usage and offer suggestions for improving its efficacy in community healthcare settings.

### Participants: Enabler

#### Training and capacity building.

Many respondents reported that the initial training that was provided prior to the eCHIS roll-out was beneficial in building their confidence as well as competence. Further, the initial face-to-face training equipped them with the practical knowledge and skills to navigate eCHIS effectively.

*Res1: “Yes, yes, I received the training on the eCHIS, it was really helpful. It was elaborate.”.*

*Res6: “Training helped me navigate the system.”*

*Res2: “After the training, I knew how to use eCHIS very well”*

The results showed that in counties where eCHIS had been used for a longer duration and users had received multiple rounds of training, they demonstrated greater confidence in using more features than counties where users received eCHIS training only once and had used eCHIS for less than one year. Further, having continuous capacity-building opportunities strengthened users’ familiarity with new updates and enhanced their ease of use with the system.

#### Supervision and support.

Enhanced supervision and support equally emerged as a key enabler of sustained eCHIS usage. Through eCHIS’s dashboard, CHAs monitored CHP performance in real time and identified areas that needed targeted support.

*Res5: “I can access the dashboard and also monitor the performance of the CHPs. From there, I can identify the weak CHPs who are not doing so well. So that I could support them.*.

*Res4: “For me ... it is easy to follow up what they are doing... I know CHP so and so has visited this household or not.”*

*Res3: “I can monitor the performance of CHPs and support weak ones.”*

Through such real-time oversight, the CHAs were able to promptly address arising gaps and provide guidance. Additionally, supervision and support weren’t reserved for formal review meetings only but were extended to immediate peer-to-peer support via digital communication platforms such as WhatsApp groups. This approach ensured CHPs had immediate access to user support and were not alone in troubleshooting challenges.

#### Motivation and incentives.

Motivation and incentives played a significant role in enhancing sustained eCHIS usage amongst frontline workers. First, the introduction of monthly stipends, combined with recognition programmes (awards for high-performing community health units (CHUs) and CHPs) boosted their morale and demonstrated that their efforts were valued. Additionally, these financial and recognition incentives made the CHPs feel appreciated and motivated them to remain active on the platform.

*Res2: “It has made my work easier so I enjoy using it.”*

*Res5: “If I stop using eCHIS it will be difficult to work.”*

*Res11: “If they can give us a certain stipend every month... then we do job.”*

Additionally, there were other practical support mechanisms that enabled consistent eCHIS usage. For example, smartphone insurance and whitelisting to ensure data submission without the need for data bundles motivated them to continue using eCHIS. Further, establishing a partnership with service providers for phone repairs ensured that technical issues with their devices could be resolved quickly at no or low cost, thereby maintaining continuity in eCHIS usage. Further, some participants reported being invited to national or county-level health events as a reward for their performance. Cumulatively, these initiatives strengthened ownership of their work and encouraged their commitment to consistently use eCHIS.

#### User engagement & confidence.

The use of eCHIS was reported to enhance CHPs’ confidence in delivering community health services during the routine household visits. Through access to real-time household data and service history, they reported feeling more prepared and authoritative when engaging the household members.

*Res1: “eCHIS has helped us a lot because when you get into the household, you know what to do”.*

*Res6: “Before we did not know what to do.....”*

Furthermore, the system’s ability to streamline task lists and provide decision-support information improved their productivity and reduced the cognitive burden of recalling household information. Concerning CHAs, their supervisory module made it easier not only to track CHPs’ performance in real time but also to quantify their supervision efforts, document, and report their supervisory work accurately.

*Res12: “My work was paper-based at first. But now that I transitioned to eCHIS, I’ve been able to do supervision that can be quantified.”*

*Res4: “If you ask me information about a household wherever I am, I can give you since it is in the phone, not like before when I had to go check the books”*.

This ability to quantify and visually track supervisory tasks appeared to reinforce accountability, enhancing their confidence that their work was being recorded and could be measured. This, in turn, encouraged them to use the system even more consistently.

#### Data use and accountability.

A sense of data ownership and data use equally emerged as another enabler towards eCHIS usage. The users felt empowered by their ability to collect, manage, and easily retrieve household data whenever needed. Through this empowerment, there was more intentional engagement with the system, thus improving their reporting habits and strengthening the alignment of their activities with the community health goals.

*Res12: “There is a time I had a gender-based violence case under my households and wasn’t able to know the details, however, I heard people mention the name of the child, I went to eCHIS, got the age of the child and was able to report the case”*.

*Res20: “My boss is able to see from the dashboard. This is what I’ve done. This is the CHP I’ve seen today.”*

In this case, having critical information at their fingertips via eCHIS enabled the CHPs to take prompt and appropriate action. Finally, the CHPs’ ability to view and display the results of their work in an easy-to-use format instilled a sense of pride and made them feel more accountable to both their supervisors and the communities they serve, hence ensuring consistent eCHIS usage.

### Participant: Barriers

#### Unequal and inadequate training.

Training was labelled as a barrier due to inconsistencies in duration and depth. Some respondents reported having been trained for only two days, and others for five days. This variation created uneven skill levels among users and left some unprepared to fully grasp how to use eCHIS.

*Res9: “The training was too short. We learnt how to enter data, but not all the other things the system can do.”.*

*Res6: And you would wish that more trainings to be done as the refresher trainings for sure.”*

This underscores the need for regular refresher training to ensure they remain proficient in using eCHIS and stay up to date with new features/updates.

#### Household resistance.

Several respondents reported that some households were reluctant to disclose sensitive information, particularly on chronic conditions or stigmatised conditions, to the CHPs which resulted to under-reporting of such critical conditions.

*Res11: “Some households are adamant to share information.”*

*Res9: “Some clients do not want to disclose sensitive information.”*

This hindered effective data collection and service delivery within such settings.

#### Financial constraints.

Although financial stipends were introduced, the respondents termed them insufficient to cover the prevailing living costs. As a result, they sought alternative income-generating activities that diverted time away from eCHIS-related activities. This resulted in missed household visits and reduced service continuity.

### Information: Enabler

In the WSF, this dimension relates to the information inserted or produced by the work system, whose availability and use drive the work processes. Participants’ lived experiences with eCHIS revealed that information-related determinants were critical to consistent eCHIS usage. In particular, the participants highlighted four key enablers in the information dimension: real-time data insights, enhanced access to information, improved data accuracy, and greater accountability through increased transparency. Each of these enablers strengthened participants’ engagement with eCHIS, thereby enhancing the system’s effectiveness in supporting community health services.

#### Real-time data insights.

The availability of real-time insights via eCHIS emerged as a key enabler for consistent use. The system provided immediate feedback on community health activities, which enabled users to respond promptly as well as plan their work more effectively. For instance, the system reminds the workers of clients due for services or flags missed visits, prompting quick follow-ups. Such real-time monitoring has enabled the CHPs to prioritise households based on current needs and address gaps before they escalate. Additionally, their supervisors can view the information almost instantly, allowing them to see which households are covered by each CHP and offer timely guidance and support. The CHPs felt that eCHIS has not only helped them in recording information but also has been used on the spot to improve care.

*Res5: “There is a time I had a gender-based violence case under my household and wasn’t able to know the details; however, I heard people mention the name of the child, I went to eCHIS, got the age of the child, and was able to report the case”*.

*Res11: “Even if I am not around, I am able to follow them up, wherever I am, so it is easy; it is just a phone call away. “Res11: I can access household data wherever I am.”*

Ultimately, this real-time information feature in eCHIS cultivated a sense of efficiency and proactivity, enabling them to consistently use it.

#### Enhanced access to information.

Improved access to health information for frontline workers was equally reported as a key enabler towards sustained eCHIS usage. The digitisation of community health records enabled them to access the health information wherever they were.

*Res17: “There are instances I am asked information about my household and am away, I normally log in to eCHIS and can respond”*.

This ready access to up-to-date information reduced the effort and time previously spent flipping through paper registers. As a result, they perceived the system as directly helpful to their work, which encouraged them to use eCHIS consistently.

#### Enhanced data accuracy and reporting.

Improvement in data accuracy and reporting was yet another key enabler of sustained eCHIS usage amongst CHPs. The transition from manual reporting to digital reporting ensured that the CHPs no longer had to contend with challenges such as arithmetic errors in tallies, illegible handwriting, or misplaced records. Once the information was captured into eCHIS, it was securely stored and easily retrievable, thus improving users’ confidence in the system’s reliability. Moreover, the platform streamlined reporting processes by enabling quick access to the aggregated data via dashboards. This functionality has facilitated the timely submission of reports and minimised delays.

*Res18: “We were recently giving vitamin A from the eCHIS I was able to pull out all the children under 5 years from the households, I visited such households to confirm if they have up-to-date issuance of vitamin A”*.

*Res2: “The data has been summarized for me, computerized summarization, meaning that is no mistake.”*

Thus, knowing that the data being input would be accurate and used for important decisions motivated the participants to engage with eCHIS regularly.

#### Accountability through enhanced transparency.

Finally, another key enabler of consistent eCHIS usage was increased accountability, fueled by data transparency. Since eCHIS data was easily aggregated and viewed at different levels of the health system, the users became more accountable in maintaining up-to-date records.

*Res:13 “I can see if I’ve visited all my households – if I miss someone, they will know from the data”*.

*Res3: “I can identify the weak CHPs who are not doing so well.”*

*Res4: “I know CHP so and so has visited this household or not.”*

Indeed, eCHIS creates a transparent record of each CHPs’ activities visible to their supervisors. Additionally, this transparency has eased the identification of coverage gaps or data entry lapses and addressed them quickly.

### Information: Barriers

#### Data synchronisation issues.

The respondents reported persistent challenges with data synchronisation on from their smartphones. The eCHIS often failed to synchronise in cases where the respondents had borrowed advanced airtime on their phones.

*Res9: Sometimes I have debt ‘Okoa’ so it doesn’t sync immediately. After I pay the Okoa debt then I can sync the records.”*

*Res19: When you are on advanced airtime ‘OKOA JAHAZI’... the whitelisting does not work.”*

Additionally, the CHPs and their supervisors identified inconsistencies between the MOH515 (summary dashboard) and the CHPs’ follow-up dashboard. It was found that the summaries generated on the two platforms did not always match, raising concerns about the data accuracy.

*Res15: “...you as a CHP have visited, let’s say 98 households in that month, another CHP visited 70, if you do an addition of all your CHPs in that month, you will always have a higher figure compared to the one in the MOH515*.

### Technology: Enablers

Within the WSF, technology focuses on the capabilities provided by the eCHIS to support or constrain work being undertaken. A number of technology-related capabilities emerged as critical enablers of sustained eCHIS usage amongst frontline health workers. These included device availability and portability, mobile accessibility and offline functionality, dashboard-enabled transparency, and a user-friendly interface.

#### Device availability & portability.

The extensive availability of smartphones, provided by the Ministry of Health with the support of various partner organisations, was cited as a key enabler to eCHIS usage. Their portability significantly reduced the burden of carrying manual records, which were often bulky and difficult to transport, especially in rural, resource-constrained environments.

*Res2: “I just walk with the phone unlike paper tools which were bulky.”*

*Res 11: “There are instances I am asked information about my households and am away, I normally log in to eCHIS and can respond. Before, it was not possible since I had to have the manual registers with me, and you can’t move with them as they are bulky.”*

*Res6: “Before we carried big books but now we carry the phone.”*

Moreover, the respondents noted that smartphones were safer, easier to carry, and more comfortable to use in the field than paper registers. In environments prone to adverse weather conditions, such as rain and flooding, these devices addressed the vulnerabilities of manual registers, which are easily damaged by environmental exposure.

#### Mobile accessibility and offline functionality.

Mobile accessibility and offline capabilities were identified as equally enabling sustained eCHIS usage. Unlike the manual registers, eCHIS enabled users to store and access information regardless of internet connectivity, a critical feature in rural areas with intermittent internet coverage. This accessibility supported uninterrupted service delivery and ensured data could be entered during household visits and synchronised later when connectivity was available. Further, the eCHIS dashboard offered immediate access to key indicators and summary data.

*Res12: “Sometimes you have to record and when you get to a place you can use eCHIS, you sych data.”*

*Res9: “I can access the dashboard and also monitor the performance of the CHPs.”.*

These capabilities streamlined task execution and reporting, enabling users to act on household-level data without delay and thereby facilitating sustained eCHIS usage.

#### Real-time usage dashboards.

The eCHIS platform introduced transparency into the supervisory module feature, which allowed supervisors to monitor their performance via real-time dashboards. These real-time dashboards displayed household visits, data completeness, and follow-up rates, enabling the supervisors to quantify their support activities and offer personalised interventions accordingly.

*Res7: “My boss can see from the dashboard. This is what I’ve done. This is the CHP I’ve seen today. These are the areas I supervise.”.*

*Res13: “We go through the dashboard, and identify some of the CHPs that are not managing the specific indicators.”*

It’s worth noting that this transparency not only facilitated targeted supervision but also equally contributed to early detection of data gaps and service delivery lapses.

*Res19: “Under community-based surveillance, I can see the cases that come up, we call them outbreaks, and new cases coming up that have not been there before.”*.

*“The MOH515 report module summarizes data for me.”*

By making CHPs’ performance and health trends visible to supervisors, the platform fostered a data-driven culture of accountability and continuous improvement toward sustained use of eCHIS.

#### User-friendly interface.

The respondents praised the eCHIS platform for its intuitive design and ease of navigation. A number of key functionalities, such as household registration and visits, task tracking, and automated reporting, were noted as having greatly reduced the cognitive burden of service delivery and encouraged consistent engagement with the system. Notably, the MOH515 reporting feature and the CHP follow-up dashboard were cited as particularly useful.

*Res4: “The reporting feature is easy to use when generating the monthly reports, which we submit to the CHAs.”*

This usability feature aligned with the established best practices in digital health design, where simplicity, clear workflows, and responsiveness are strongly associated with usage.

### Technology: Barriers

#### Infrastructural issues.

Although the respondents were provided with smartphones to support the eCHIS, they faced several device challenges, including frequent breakdowns,phone charging, theft, login issues, and network connectivity issues that affected eCHIS usage.

*Res11: “When the phone stops working, I have to wait for weeks before it can be replaced. In that time, I cannot enter data.”*.

*Res13: “Sometimes it takes long to upload due to network.”*

*Res6: “When there is no network syncing becomes a problem.”*

*Res7: “Sometime maybe the battery has gone low or some charging system or the screen is not okay.”*

This results in some CHPs reverting to paper-based data capture, and the data captured in this way fails to be entered into eCHIS and cannot therefore be relied upon during decision-making.

#### Mandatory fields.

The respondents expressed frustration with some mandatory fields in the eCHIS household registration form, such as phone numbers and national identification numbers, which couldn’t be bypassed in households where the head lacked a phone number or even an identification card, and thus the registration couldn’t be completed digitally.

*Res7: “Sometimes the head of the household has no phone or ID. The system refuses to save, so we have to go back to the book.”*.

This resulted in inconsistent eCHIS usage as users reverted to manual registers, disrupting the digital flow of household registrations.

### Processes: Enablers

Within the WSF, the process refers to the set of interrelated activities that are aimed at delivering community health services. The household visit was the central process through which frontline workers delivered community health services. Below, several process-related capabilities emerged as critical enablers of sustained eCHIS usage amongst frontline health workers. These included streamlined and standardised workflows, built-in decision-support tools, real-time task management, data-driven monitoring, and outcome orientation.

#### Streamlined processes for data collection.

eCHIS significantly simplified the processes associated with household registration and follow-up, which previously were manually undertaken via ministry-approved reporting books. The respondents reported that the digitisation of the workflows had enabled them to undertake their assigned tasks more efficiently, reducing duplication and administrative fatigue, allowing CHPs to manage their tasks more effectively and efficiently. Through eCHIS, it became easy to record household demographics, deliver services, conduct follow-ups, and plan future visits, all within a single platform.

*Res11: “ But with eCHIS, I just wake up, go to my task, I know that I should follow up this person. I made a referral here*.

*Res4: “We were recently giving vitamin A, from the eCHIS I was able to pull out all the children under 5 years from the households. I visited such households to confirm if they have up-to-date issuance of vitamin A”*.

This ease of documentation enabled the CHPs to maintain momentum during household visits, eliminating the need to return to a central point for data aggregation and reporting.

#### Real-time task management and service prioritisation.

The respondents reported that the eCHIS task list feature and automated alerts helped them prioritise their daily activities. For instance, the system could generate reminders for follow-ups or missed household visit activities, allowing them to act quickly and reduce service delays.

*Res11: “The system shows me what I have not done. If I skipped a household or forgot a visit, it reminds me before I go far.”*

*Res5: “The phone reminds me to do follow-up.”*

#### Automated summaries and reporting.

eCHIS streamlined workflows by automating the generation of monthly reports. The CHPs no longer manually compiled the statistics from manual records. Instead, through the MOH515 feature within the platform, it was now possible to access community data and generate reports by availing summarised data on different health indicators.

*Res14 “Reporting is now very easy. I don’t have to count everything at the end of the month. The system shows me the totals, and I just submit.”*.

### Process: Barriers

#### Missed/inconsistent household visits.

The respondents reported that heavy workloads stemming from the high number of assigned households, competing priorities, and resource constraints often led to delayed or missed household visits. Due to irregular visits, they were unable to update household information in eCHIS.

*Res16: “There are times I don’t reach all my households. When that happens, the data in eCHIS is incomplete.”*.

*Res6: “Now we have a flood. And the whole village is flooded, am not able to visit households.”*

#### Delayed eCHIS updates.

Some regions experienced discrepancies in the data summaries presented on the MOH515 dashboard, leading to confusion and mistrust in the reported data. This inconsistency hindered the effective monitoring and evaluation of community health services. The respondents affirmed inconsistencies in the data summaries presented on the MOH515 dashboard as they failed to match the summations availed on the CHP follow-up dashboard.

*Res4: “Again, as I said, on the performance, it should be updated every day. It doesn’t show on my side immediately.”*

*Res12: “...you have a CHP, visited, let’s say 98 households in that month, another CHP visited 70, if you do an addition of all your CHPs in that month, you will always have a higher figure compared to the one in the MOH515.”*

As shown in Table 3, there was no single WSF dimension that holistically accounted for sustained eCHIS usage. Instead, the findings revealed that determinants affecting eCHIS usage were shaped by the alignment of the four work system dimensions.

**Table 3:** Summary of enablers and barriers to consistent eCHIS usage.

WSF Domain	Main Enablers	Main Barriers	Implication for Consistent eCHIS Usage
Participants	Training and capacity building; supervision and peer support; motivation and incentives; User Engagement and Confidence; data ownership and accountability	Unequal and inadequate training; financial constraints; household resistance to disclosure of sensitive information.	Shapes users’ capability, motivation, and willingness to incorporate eCHIS into routine community health work.
Information	Real-time data insights; enhanced access to household information; enhanced data accuracy and reporting; greater data transparency	Data synchronisation problems; inconsistencies between MOH515 and CHP follow-up dashboard summaries	Determines whether eCHIS information is available, reliable, and useful for service delivery, monitoring, and decision-making.
Technology	Availability and portability of smartphones; mobile accessibility and offline functionality; real-time usage dashboards; user-friendly interface	Device breakdowns and replacement delays; charging problems; theft; login difficulties; poor network connectivity; rigid mandatory data fields	Enables or interrupts users’ access to and execution of eCHIS functions during routine community health service delivery.
Processes	Streamlined processes for household registration and follow-up; real-time task management and service prioritisation; automated summaries and reporting	Missed or inconsistent household visits due to workload; delayed eCHIS/dashboard updates	Determines whether community health activities are actually performed and documented through eCHIS as part of routine service delivery.

## Discussion

This study examined the enablers and barriers to eCHIS usage among healthcare workers in Kenya through the lens of the WSF. These study findings demonstrate that eCHIS usage is not influenced by technological factors in isolation but is shaped by the interaction between participants, information, technology, and processes. This is consistent with Sittig and Singh [21] argument that several interdependent social and technical dimensions influence how health information technologies operate. Our findings extend this argument to community-based eCHIS by capturing a comprehensive picture of the socio-technical determinants influencing eCHIS usage. For example, even with functional device availability (technology), household visits (process) were inconsistent, resulting in incomplete data capture, and thus technology alone could not guarantee eCHIS usage. Additionally, even when user training enhanced their confidence in using eCHIS, synchronisation issues (technology) and data inconsistencies (information) equally affected consistent eCHIS usage. Overall, the findings demonstrate that while frontline workers appreciate the value of eCHIS in improving community health service delivery, multiple interlinked barriers continue to constrain consistent usage. Several findings, such as device breakdown, network challenges, and financial constraints, were expected to have been captured equally in other related studies.

The role of adequate and continuous training observed in this study aligns with earlier works on eCHIS usage in Ethiopia [7,22],on EMRs usage in Kenya [12] and on eCHIS implementation in Kenya [23], which found inadequate training as a key barrier. Consistent with the above evidence, these findings reinforce our observation that subsequent refresher training is needed to foster sustained eCHIS usage. The supervisory role of eCHIS dashboards observed in this study was consistent with evidence from other digital community health systems. In Busia County, real-time dashboards enabled the supervisors to remotely monitor CHPs’ performance and offer timely support [24]. Equally, personalised performance feedback arising from dashboard-supported supervision was found to increase the number of household visits undertaken by CHPs [25]. These findings support the idea that real-time dashboards have the potential to create a data-driven culture of accountability and continuous improvement towards sustained use of eCHIS. Financial incentive was found to be both an enabler and a barrier affecting CHPs’ ability to actively use eCHIS. Studies have shown that financial incentives boost the morale of the CHPs, supporting our finding that stipends offer motivation for continued engagement with eCHIS [26].In yet another study, financial incentives were found to enhance the performance of CHPs, increase household visits and referrals [27]. However, three findings that extend the existing literature were novel in this study. Firstly, CHPs derived a strong sense of pride and professional identity arising from data ownership, describing how eCHIS enhanced their confidence and empowered them to respond effectively to cases. At the same time, the household’s resistance to sharing sensitive information emerged as a critical socio-cultural barrier influencing eCHIS usage. This has a direct impact on data quality, as incomplete disclosure leads to under-reporting and ultimately misrepresents true usage. It equally suggests that eCHIS may be perceived as a surveillance tool if trust is not properly established. Secondly, whereas incentives such as stipends and recognition supported usage, the respondents stressed that they remain insufficient to offset the current economic pressure and thus the need for more enhanced financial and non-financial support. Thirdly, the study found that consistent usage depends on aligning eCHIS with the frontline processes, as technology alone cannot independently generate consistent usage when the process (household visits), which is the main point of service contact, is skipped, disrupted, or undertaken outside the system. Without household visits being captured via eCHIS, essential service outcomes such as WASH assessment and maternal follow-up cannot be systematically managed.

The strength of this study lies in its application of WSF, which ensured that enablers and barriers were examined holistically across socio-technical dimensions. Nevertheless, there were several limitations to this study. First, the study relied solely on participants’ self-reported experiences, which may be subject to recall bias. Although the qualitative approach offered rich insights into the lived experiences of frontline workers, the study did not have an opportunity to triangulate these findings with system usage logs to provide objective measures of actual system usage. However, the qualitative findings offered rich contextual evidence on determinants of eCHIS usage. Secondly, the study was conducted only in three counties, and while they provided rich insights, the findings may not be fully generalisable to all counties. Thirdly, the study focused on users directly interacting with eCHIS and thus failed to include perspectives from higher-level health system managers, system developers, or policymakers, whose additional insights may have influenced the results. Future studies should adopt a mixed-method approach where qualitative insights can be triangulated with system usage data and service delivery indicators to provide a more comprehensive understanding of how such determinants influence the relationship between eCHIS usage and health outcomes.

Despite these limitations, our findings offer contextual and socio-technical insights with direct implications for different stakeholders. Firstly, digital health interventions must be viewed as socio-technical systems rather than purely technological solutions, and their use must be evaluated through the same lens. Our study offers crucial information that will be helpful to decision-makers at different levels, including the Ministry of Health (MOH), county leadership, and local implementing partners, for successful eCHIS utilisation. There’s a need to institutionalise refresher training and embed data protection protocols to build both user competence and community trust. Equally, for the county leadership, through the assigned focal persons, they must establish a mechanism to strengthen supervision and provide technical and infrastructural support to ensure consistent eCHIS usage. Thirdly, frontline workers must undertake community sensitization and trust-building initiatives during routine service delivery to ensure the communities understand the purpose of data collection and how confidentiality is maintained. This is because trust between frontline workers and community members is key to ensuring data quality. Finally, implementers should be able to refine the system to align with the realities of data field elements across different regions, for example, removing rigid, mandatory fields that exclude vulnerable households and reducing duplicate reporting burdens.

## Conclusion

The study set out to evaluate the enablers and barriers to consistent eCHIS usage in Kenya, guided by the WSF as our analytical lens. Whereas significant investments have been made in digital health systems, the study demonstrates that system availability alone does not guarantee consistent eCHIS usage. On the contrary, eCHIS usage is shaped by interactions among participants, information, processes, and technology. eCHIS has the potential to strengthen community health service delivery. However, its consistent usage depends on addressing the interlinked socio-technical barriers. By applying the WSF, the study contributes to a more holistic and realistic explanation of why digital health systems usage remains low. Addressing these findings will be critical to enhancing eCHIS usage and to offering reusable lessons for other LMICs seeking to yield the benefits of digital health investments.
