# Supplementary material for: Enablers and barriers to Electronic Community Health Information System (eCHIS) usage in Kenya: A Work System Framework Analysis of frontline health worker experiences
Source: PLoS One. 2026 Oct 8;21(10):e0358994. doi: 10.1371/journal.pone.0358994 (PMC13649138; doi:10.1371/journal.pone.0358994)
Supplement: Supplementary file 1 [file pone.0358994.s003.pdf]

Dear Reviewer,

RE: SUBMISSION OF REVISED MANUSCRIPT

We sincerely thank you for the careful evaluation of our manuscript[Enablers and Barriers to Electronic Community Health Information System (eCHIS) Usage in Kenya: A Work System Framework Analysis of Frontline Health Worker Experiences] and for the constructive comments and recommendations provided. We appreciate the opportunity to revise the manuscript and believe that the revisions undertaken have substantially improved its clarity, methodological reporting, presentation of findings, and overall contribution.

We have carefully considered and addressed all the comments raised by the reviewers. A detailed point-by-point response has been prepared, indicating how each comment was addressed and where the corresponding changes have been made in the revised manuscript.

Among the key revisions, we have strengthened the Introduction to provide clearer theoretical and empirical justification for examining sustained eCHIS usage and its socio-technical determinants. We have also clarified the application of the Work System Framework as the analytical lens guiding the study.

The Results section has been re-organised to begin with participants' characteristics before presenting the thematic findings. We have additionally included a summary table organising the principal enablers and barriers according to the four Work System Framework domains—participants, information, technology, and processes—to provide readers with a concise overview of the findings before the detailed thematic presentation.

The Discussion has also been strengthened by engaging more extensively with relevant eCHIS, digital health, and health information systems literature. The revised discussion more clearly distinguishes findings that are consistent with previous research, those that extend existing evidence, and the contribution of the Work System Framework in explaining how factors across participants, information, technology, and processes interact to shape consistent eCHIS usage.

Thank you for your time and consideration.

Yours sincerely,

Michael Kagiri

ABSTRACT		
**REVIEWERS COMMENT**	**ACTION TAKEN**	**LINE NUMBER**
Drop (UHC), as it appears only once in the Abstract	UHC was removed and the statement remained only with universal health coverage	On the first paragraph
Similarly, spell out all abbreviations (e.g., Community Health Promoters [CHPs]) the first time they appear.	The abbreviations CHPs, CHAs were rewritten in full in the methods section of abstract	On the methods section
In the final sentence of the conclusion, remove the phrase "that cannot be generalised from Ethiopia's experience." The conclusion should focus on the study findings and implications rather than making comparisons with other settings.	This has been removed and linked back to study findings and implications	Conclusion section of abstract
**INTRODUCTION**		
Consider restructuring the introduction to improve its logical flow: Paragraph 1: Present the problem statement by combining the current first and second paragraphs into a concise overview. Paragraph 2: Summarize what is currently known about the barriers and facilitators to eCHIS implementation, identify the knowledge gaps, and explain what remains unknown. Paragraph 3: Introduce the Kenyan Community Health	Reworked and made more comprehensive	

Program and the eCHIS intervention. Paragraph 4: Present the rationale and significance of the study, followed by Paragraph 5 the study objectives.		
Paragraph 3 is long. Try to trim or make 2 paragraphs	This was broken down into smaller paragraphs	
The fourth paragraph (Lines 65–71) largely repeats issues already presented in the second paragraph. Instead, dedicate this paragraph to clearly explaining the rationale, significance, and contribution of the study.	Amended from line 99 to 126 to explain the significance and contribution of the study	99-126
Several citations are not integrated clearly into the narrative: Line 12: The sentence beginning "[3] affirms that..." is unclear. Either mention the author's name or rewrite the sentence. The same issue occurs in: Line 18 ("For instance, a study by [4]..."); Line 23 ("...consistent with [5] findings..."); Line 66 ("A study by [1]..."); Line 130 ("...outlined by [13]."). Revise these sentences so that citations are incorporated appropriately.	Amended to i.e a study by Ngugi……………	
Line 33: Provide the equivalent value in USD for KSh 2,500, indicating the exchange rate or base year used.	Amended to include the amount USD 19.3 as well	16

Ensure abbreviations are introduced only once: Line 43: Do not abbreviate Universal Health Coverage again if it has already been introduced. Line 79: Spell out Work System Framework (WSF) at first mention, and then use WSF consistently thereafter. Consequently, the full term does not need to be repeated in Lines 85 and 88.	Removed the abbreviation UHC from universal health coverage.<br>All instances of work system framework in full were replaced with WSF in subsequent sections.	42<br>WSF amended on Line 102 and 107
**METHODS**		
Line 97, change "ECHIS" to "eCHIS"	This has been amended to eCHIS as shown on	122
Line 101, replace the word "was" with "were"	The whole sentence was rewritten to "The study was conducted in three counties (Kakamega, Kisumu, and Migori), in Kenya, in April 2024, where the Electronic Community Health Information System (eCHIS) had been implemented and had been in<br>use for more than 17 months.	126-128
Line 102 provide additional description of the study settings	The following additional information was added "The study was conducted in three counties (Kakamega, Kisumu, and Migori), in Kenya, in April 2024, where the Electronic Community Health Information System (eCHIS) had been implemented and had been in use for more than 17 months. The counties were purposively selected because they were among the early users of the eCHIS system and had been using it for a longer period, making them suitable for studying factors influencing sustained system use rather than initial utilisation. Further, participants assigned to respective community health units (CHUs) within each county were selected based on CHUs' linkage to the Kenya Essential Package for Health (KEPH) Level 2, Level 3, and Level 4 health facilities.	126-134

Line 108, replace (9–17) with 9-17	Amended to 9-17	Line 143
Line 109-110, don't redefine Community Health Promoters(CHPs) and Community Health Assistants (CHAs). Please check these throughout the manuscript	Amended to only have it defined once and the abbreviation used elsewhere. Rechecked the whole manuscript	
The data collection period appears to be March 6, 2024, to April 10, 2024 (Line 119). Please revise the study period reported in Line 102 to ensure consistency.	This was amended in line 126 to be consistent with line 154-156	154-156
Remove the sentences in Lines 112–113 describing IRB approval, as ethical approval is typically reported elsewhere. Instead, include details such as the language(s) used for data collection and where interviews were conducted (e.g., participants' homes or workplaces).	This was reworked	
**FINDINGS**		
Begin the Results section with participants' characteristics before presenting the thematic findings	This was amended by add the participant characteristics and the table	193-203
Consider including a summary table that organizes the identified barriers and enablers according to each analytic domain. This would help readers quickly understand the main findings.	We have added a summary table at the beginning of the thematic findings to provide readers with an overview of the principal enablers and barriers identified in the study. Consistent with the analytical approach used in the study, the findings are organized according to the four Work System Framework domains: participants, information, technology, and processes. The table summarizes the major enablers and barriers within each	From 552

	domain before these findings are subsequently presented in greater detail and supported with participants' accounts.	
**DISCUSSION**		
Line 451: Do not redefine Work System Framework (WSF) if it has already been introduced earlier in the manuscript.	Removed to only with WSF	Line 536
The third paragraph of the Discussion is lengthy. Consider splitting it into two paragraphs, with one dedicated specifically to the study's strengths and limitations.	This has since been addressed by having a paragraph on strengths and limitations	From line 589
Revisit the final paragraph. Avoid using the term "generalizable" in the conclusion. In addition, the paragraph states that the study provides four insights, but only three are actually presented. Please revise for consistency.	Amended	Line 637-640
