# Supplementary material for: Enablers and barriers to Electronic Community Health Information System (eCHIS) usage in Kenya: A Work System Framework Analysis of frontline health worker experiences
Source: PLoS One. 2026 Oct 8;21(10):e0358994. doi: 10.1371/journal.pone.0358994 (PMC13649138; doi:10.1371/journal.pone.0358994)
Supplement: Supplementary file 2 [file pone.0358994.s002.docx]

Dear Editor and Reviewers,

We sincerely thank you for the careful review of our manuscript and for the constructive comments and suggestions. We have carefully revised the manuscript to address all comments raised. The revisions have significantly improved the clarity, methodological transparency, and theoretical contribution of the paper. Below is a detailed, point-by-point response to each reviewer comment and a description of the changes made in the revised manuscript.

**Responses to Reviewer #1**

**Comment 1: Limited number of quotes used and repeated quotes; concern whether all interviews were analyzed.**

**Response:**
We thank the reviewer for this important observation. We have revisited the interview transcripts and conducted a more comprehensive analysis to ensure that all interviews were fully analyzed. The Results section has been revised to include additional quotes from a wider range of participants across all study regions to better reflect the diversity of participant experiences and to improve the depth and richness of the qualitative findings. Repetitive quotes have been removed and replaced with new quotes to ensure broader representation of participant perspectives.

**Changes made:** *Results section revised to include more participant quotes and broader representation (Pages 6–13).*

**Comment 2: Conclusions in the abstract do not reflect results and read like recommendations; inconsistency regarding Ethiopia study.**

**Response:**
Thank you for this observation. The abstract has been rewritten to ensure that the conclusions reflect the actual findings of the study rather than recommendations. We also revised the section discussing findings from Ethiopia to ensure consistency between the abstract and the results section. The abstract now clearly summarizes the research problem, methods, key findings, and contribution of the study.

**Changes made:** Abstract rewritten.

The Electronic Community Health Information System (eCHIS) is Kenya’s flagship Digital health solution rolled out to strengthen community health service delivery and accelerate progress toward achieving universal health coverage (UHC). Despite its widespread rollout, evidence from global health initiatives affirms that the mere presence of a system doesn’t guarantee meaningful use. Consistent eCHIS usage by frontline health workers remain a critical determinant of realising anticipated benefits, underscoring the need to understand the context-dependent enablers and barriers influencing such consistent usage based on users’ lived experiences.

Methods: We employed a qualitative phenomenological design in three counties, conducting 22 interviews with CHPs(n=12), CHAs(n=6), and county focal persons (n=4). The qualitative data were transcribed and analysed thematically using NVivo, guided by the Work System Framework (WSF), which examined usage across four dimensions: participants, information, technology, and processes.

Results: The findings showed that sustained eCHIS usage is influenced by a set of interrelated socio-technical enablers and barriers across the WSF dimensions. The key enablers included incentives, supportive supervision, training opportunities, real-time data access, improved accuracy, device portability, transparency, and data ownership. The major barriers included inadequate and unequal training, household resistance to disclosing sensitive information, financial constraints, device failures, data synchronisation issues, missed household visits due to environmental challenges, and delayed system updates.

Conclusion: The study’s contribution was to show that sustained system use is shaped by interactions among work system dimensions rather than by technology availability alone. Through the WSF, the study provides a holistic understanding of the socio-technical determinants that influence the sustained use of national digital health systems. The findings affirm the need for refresher training, infrastructure support workflow alignment, and community trust to ensure sustained use of digital health systems in low-resource settings.

**Comment 3: Ethical considerations should clearly state that real names and identifying information were not used.**

**Response:**
We appreciate this comment and have revised the Ethical Considerations section to explicitly state that no real names or identifying information were collected, recorded, or reported in the study, and that participant confidentiality and anonymity were maintained throughout the research process.

**Changes made:** Ethical Considerations section revised (Line 161-line 169).

*Ethical approval was obtained from Chuka University institutional review board (ref:NACOSTI/NBC/AC-0812). Participants received their written informed consent in a confidential environment. They were informed about the study's benefits, hazards, confidentiality measures, and the right to withdraw without justification. In order to ensure confidentiality and anonymity, the participants were assigned unique codes i.e Res1, Res2, Res3 and the quotes were anonymised to prevent direct or indirect identification of the participants. The study ensured that real names, facility names, or identifiable personal information were not recorded in the transcripts. Thereafter, each transcript was assigned a unique code.*

**Comment 4: Typographical errors and incomplete sentences.**

**Response:**
We have carefully proofread the manuscript and corrected all typographical errors, grammatical issues, and incomplete sentences identified in the reviewer’s comments.

***Changes made:*** *Manuscript edited throughout.*

**Responses to Reviewer #2**

**Comment 1: Coverage of Work System Framework constructs; infrastructure issues such as electricity and network connectivity should be discussed.**

**Response:**
Thank you for this important suggestion. We have revised the Findings and Discussion sections to explicitly include infrastructure-related factors such as electricity access for charging mobile phones and network connectivity as part of the Technology dimension of the Work System Framework. These factors are now clearly discussed as key determinants influencing eCHIS usage.

**Changes made:** *Findings and Discussion sections revised to include infrastructure-related issues (Pages 11 from line 449 to 461).*

*Discussion section on page 13 from line 553*

**Comment 2: Summarize participants’ basic information/characteristics in a table.**

**Response:**
We have added a table summarizing participant characteristics, including role (CHA/CHP), years of experience, county, and duration of eCHIS use, to provide better context for the study sample.

**Changes made:** *New table added in Methods section (Page 5 from line 143).*

**Comment 3: Terminology consistency — ensure “eCHIS” is used consistently.**

**Response:**
*We have reviewed the manuscript and ensured that the term “eCHIS” is used consistently throughout the manuscript.*

***Changes made:*** *Terminology has been standardized throughout manuscript.*

**Comment 4: Health system context — briefly describe Kenya’s health system structure in Methods section.**

**Response:**
*We have added a subsection in the Introduction section describing Kenya’s community health system structure, including the role of Frontline Community Health Workers, Community Health Assistants, and how eCHIS is used within the community health system.*

***Changes made:*** *Health system context added in Introduction section (Page 1 from line 2 to line 17).*

*Kenya’s health system is a devolved function structured into six levels: community, dispensary, health centre, sub-county hospital, county referral hospital, and national referral hospitals. Of the six levels, Level 1 facilities (community health units) are responsible for community-level services, delivered by frontline community health promoters (CHPs), supervised by community health assistants (CHAs), and coordinated at the county level by community health focal persons.* *In Kenya, the backbone of community health services began with frontline community health workers (CHWs), who were selected by their communities and trained to provide basic healthcare services, health education, and referrals to local households without any formal compensation. Despite this, many exceeded their expected two-hour commitment, often juggling health duties alongside other jobs and family commitments. The CHWs were required to manually record and input health data into large, cumbersome ministry-approved books. However, this changed in 2017, where their role evolved into that of CHPs, accompanied by a monthly stipend of Ksh 2,500 to support their work. Starting 2021, the government, with the assistance of Living Goods, a non-governmental organization (NGO), provided the CHPs with smartphones that enabled them to upload household health data directly to the eCHIS .*

**Comment 5: Workforce terminology — use “Frontline Community Health Workers” consistently.**

**Response:**
*We have revised the manuscript to consistently use the term “Frontline Community Health Workers” when referring to CHPs in Line 5.*

***Changes made:*** *Terminology revised throughout manuscript.*

**Comment 6: Geographic scope — number of regions included should be clearly stated in Methods.**

**Response:**
We have revised the Methods section to clearly state the number of counties included in the study and explain the selection criteria for these counties.

**Changes made:** Methods section revised (Page 4) from line 126 to line 146.

*The counties were purposively selected because they were among the early users of the eCHIS system and had been using it for a longer period, making them suitable for studying factors influencing sustained system use rather than initial utilisation. Further, participants assigned to respective community health units (CHUs) within each county were selected based on CHUs' linkage to the Kenya Essential Package for Health (KEPH) Level 2, Level 3, and Level 4 health facilities.*

**Comment 7: Discussion section is brief and should be expanded; community resistance should be discussed further.**

**Response:**
We have expanded the Discussion section to provide deeper interpretation of the findings and their implications for digital health implementation and policy. We also expanded the discussion on community resistance to disclosing sensitive information and discussed its implications for data quality, service delivery in eCHIS.

**Changes made:** Discussion section expanded (Pages 12).

*At the same time, the household's resistance to sharing sensitive information emerged as a critical social-cultural barrier influencing eCHIS usage. This has a direct impact on data quality, as incomplete disclosure leads to under-reporting and ultimately misrepresents true usage. It equally suggests that eCHIS may be perceived as a surveillance tool if trust is not properly established.*

**Additional Changes Made to the Manuscript**

In addition to addressing reviewer comments, we also made the following improvements:

- Added a **Study Limitations** section.

*Nevertheless, there were several limitations to this study. First, the study relied solely on participants' self-reported experiences, which may be subject to recall bias. Although the qualitative approach offered rich insights into the lived experiences of frontline workers, the study did not have an opportunity to triangulate these findings with system usage logs to provide objective measures of actual system usage. However, the qualitative findings offered rich contextual evidence on determinants of eCHIS usage. Secondly, the study was conducted only in three counties, and while they provided rich insights, the findings may not be fully generalizable to all counties. Thirdly, the study focused on users directly interacting with eCHIS and thus failed to include perspectives from higher-level health system managers, system developers, or policymakers, whose additional insights may have influenced the results. Future studies should adopt a mixed-method approach where qualitative insights can be triangulated with system usage data and service delivery indicators to provide a more comprehensive understanding of such determinants influence the relationship between eCHIS usage and health outcomes.*

**Conclusion**

We are grateful for the insightful comments provided by the editor and reviewers. We believe that addressing these comments has significantly improved the quality, clarity, and contribution of the manuscript. We hope that the revised manuscript is now suitable for publication.

Thank you for your time and consideration.

Yours sincerely,
**Michael Kagiri**
**University of Nairobi.**
**Michael.kagiri@dkut.ac.ke**
