# Supplementary material for: Enablers and barriers to Electronic Community Health Information System (eCHIS) usage in Kenya: A Work System Framework Analysis of frontline health worker experiences
Source: PLoS One. 2026 Oct 8;21(10):e0358994. doi: 10.1371/journal.pone.0358994 (PMC13649138; doi:10.1371/journal.pone.0358994)
Supplement: Peer review history

# Peer review: Decision Letter 0

**DOI:** 10.1371/journal.pone.0358994.r001

**Authors:** Gizachew Tiruneh

21 Jan 2026

Dear Dr. KAGIRI,

==============================

**Please review the comments provided by reviewers carefully and addressed them accordingly.** plosone@plos.org. When you're ready to submit your revision, log on to https://www.editorialmanager.com/pone/ and select the 'Submissions Needing Revision' folder to locate your manuscript file.

- A letter that responds to each point raised by the academic editor and reviewer(s). You should upload this letter as a separate file labeled 'Response to Reviewers'.
- A marked-up copy of your manuscript that highlights changes made to the original version. You should upload this as a separate file labeled 'Revised Manuscript with Track Changes'.
- An unmarked version of your revised paper without tracked changes. You should upload this as a separate file labeled 'Manuscript'.

If applicable, we recommend that you deposit your laboratory protocols in protocols.io to enhance the reproducibility of your results. Protocols.io assigns your protocol its own identifier (DOI) so that it can be cited independently in the future. For instructions see: https://journals.plos.org/plosone/s/submission-guidelines#loc-laboratory-protocols. Additionally, PLOS ONE offers an option for publishing peer-reviewed Lab Protocol articles, which describe protocols hosted on protocols.io. Read more information on sharing protocols at https://plos.org/protocols?utm_medium=editorial-email&utm_source=authorletters&utm_campaign=protocols.

We look forward to receiving your revised manuscript.

Kind regards,

Gizachew Tadele Tiruneh, Ph.D.

Academic Editor

PLOS One

**Journal Requirements:**

1. When submitting your revision, we need you to address these additional requirements.

Please ensure that your manuscript meets PLOS ONE's style requirements, including those for file naming. The PLOS ONE style templates can be found at

https://journals.plos.org/plosone/s/file?id=wjVg/PLOSOne_formatting_sample_main_body.pdf and

https://journals.plos.org/plosone/s/file?id=ba62/PLOSOne_formatting_sample_title_authors_affiliations.pdf

2. We note that you have indicated that there are restrictions to data sharing for this study. For studies involving human research participant data or other sensitive data, we encourage authors to share de-identified or anonymized data. However, when data cannot be publicly shared for ethical reasons, we allow authors to make their data sets available upon request. For information on unacceptable data access restrictions, please see http://journals.plos.org/plosone/s/data-availability#loc-unacceptable-data-access-restrictions.

Before we proceed with your manuscript, please address the following prompts:

a) If there are ethical or legal restrictions on sharing a de-identified data set, please explain them in detail (e.g., data contain potentially identifying or sensitive patient information, data are owned by a third-party organization, etc.) and who has imposed them (e.g., a Research Ethics Committee or Institutional Review Board, etc.). Please also provide contact information for a data access committee, ethics committee, or other institutional body to which data requests may be sent.

b) If there are no restrictions, please upload the minimal anonymized data set necessary to replicate your study findings to a stable, public repository and provide us with the relevant URLs, DOIs, or accession numbers. Please see http://www.bmj.com/content/340/bmj.c181.long for guidelines on how to de-identify and prepare clinical data for publication. For a list of recommended repositories, please see https://journals.plos.org/plosone/s/recommended-repositories. You also have the option of uploading the data as Supporting Information files, but we would recommend depositing data directly to a data repository if possible.

Please update your Data Availability statement in the submission form accordingly.

3. When completing the data availability statement of the submission form, you indicated that you will make your data available on acceptance. We strongly recommend all authors decide on a data sharing plan before acceptance, as the process can be lengthy and hold up publication timelines. Please note that, though access restrictions are acceptable now, your entire data will need to be made freely accessible if your manuscript is accepted for publication. This policy applies to all data except where public deposition would breach compliance with the protocol approved by your research ethics board. If you are unable to adhere to our open data policy, please kindly revise your statement to explain your reasoning and we will seek the editor's input on an exemption. Please be assured that, once you have provided your new statement, the assessment of your exemption will not hold up the peer review process.

4. Please amend either the title on the online submission form (via Edit Submission) or the title in the manuscript so that they are identical.

5. Please amend the manuscript submission data (via Edit Submission) to include author Dr. Maureen Kimani.

6. If the reviewer comments include a recommendation to cite specific previously published works, please review and evaluate these publications to determine whether they are relevant and should be cited. There is no requirement to cite these works unless the editor has indicated otherwise.

7. Please review your reference list to ensure that it is complete and correct. If you have cited papers that have been retracted, please include the rationale for doing so in the manuscript text, or remove these references and replace them with relevant current references. Any changes to the reference list should be mentioned in the rebuttal letter that accompanies your revised manuscript. If you need to cite a retracted article, indicate the article’s retracted status in the References list and also include a citation and full reference for the retraction notice.

[Note: HTML markup is below. Please do not edit.]

Reviewers' comments:

**Comments to the Author**

1. Is the manuscript technically sound, and do the data support the conclusions?

Reviewer #1: Yes

Reviewer #2: Yes

**********

2. Has the statistical analysis been performed appropriately and rigorously?

Reviewer #1: N/A

Reviewer #2: N/A

**********

3. Have the authors made all data underlying the findings in their manuscript fully available?

The PLOS Data policy

Reviewer #1: No

Reviewer #2: No

**********

4. Is the manuscript presented in an intelligible fashion and written in standard English?

Reviewer #1: Yes

Reviewer #2: Yes

**********

**Reviewer #1:**  This qualitative study addresses an important area related to electronic health information systems for community health workers. However, several issues require attention:

Although the authors report conducting more than 22 interviews, quotes from only a limited number of participants (1–4 from Kisumu, 1–4 from Migori, and 1–4 from Kakamega) are repeatedly used across multiple sections, with the same quotes appearing in two places. This raises concerns about whether all interviews were adequately analyzed. The limited use of quotes also restricts the depth, variation, nuance, and richness of insights derived from the data. I recommend that all interviews be analyzed and that the results section be revised accordingly.

The conclusions presented in the abstract do not adequately reflect the study results and instead read more like recommendations. In addition, evidence cited from Ethiopia is described as consistent with the findings in the results section but is presented as divergent in the abstract, which requires revision for clarity and consistency.

Under the ethical considerations section, a clear statement should be included assuring that real names or any information that could directly or indirectly identify participants were avoided, in accordance with ethical research standards.

There are several typographical errors and incomplete sentences, which I have highlighted in the comments for the authors’ consideration.

**Reviewer #2:**  Dear Editor,

Thank you for inviting me to review this manuscript. The paper is well written and addresses a highly relevant topic. I commend the authors for their dedication to conducting this study with strong methodological rigor and for generating critical findings that have the potential to significantly contribute to the scale-up and sustained use of eCHIS.

I have a few comments for the authors’ consideration, as outlined below:

Coverage of WSF constructs: While the study addresses several important components of the WSF, it is unclear whether all key constructs were comprehensively examined. For example, infrastructure-related issues such as access to electricity for charging mobile phones and network connectivity are critical for digital health implementation and should be explicitly discussed.

Please summarize the participants basic information/characteristics in Table

Terminology consistency: Please ensure that the term “eCHIS” is used consistently throughout the manuscript.

Health system context: To support readers who may be unfamiliar with the study setting, I recommend briefly describing Kenya’s health system structure in the Methods section to provide clearer contextual understanding.

Workforce terminology: Please consistently use the term “Frontline Community Health Workers” throughout the manuscript.

Geographic scope: The number of regions included in the study should be clearly stated in the Methods section to enhance transparency regarding the study’s geographic coverage.

Discussion section: The Discussion section is relatively brief and would benefit from further expansion. I encourage the authors to elaborate on the key findings and their programmatic and policy implications. In addition, community resistance to disclosing sensitive information was identified as a barrier; this is an important finding that warrants deeper discussion, particularly regarding its implications for implementation, data quality, and future scale-up of eCHIS.

Overall, this is a valuable contribution to the digital health and community health systems literature, and addressing the points above would further strengthen the manuscript.

**********

what does this mean?). If published, this will include your full peer review and any attached files.

If you choose “no”, your identity will remain anonymous but your review may still be made public.

**Do you want your identity to be public for this peer review?**  For information about this choice, including consent withdrawal, please see our Privacy Policy

Reviewer #1: No

Reviewer #2: No

**********

[NOTE: If reviewer comments were submitted as an attachment file, they will be attached to this email and accessible via the submission site. Please log into your account, locate the manuscript record, and check for the action link "View Attachments". If this link does not appear, there are no attachment files.]

To ensure your figures meet our technical requirements, please review our figure guidelines: https://journals.plos.org/plosone/s/figures

You may also use PLOS’s free figure tool, NAAS, to help you prepare publication quality figures: https://journals.plos.org/plosone/s/figures#loc-tools-for-figure-preparation.

NAAS will assess whether your figures meet our technical requirements by comparing each figure against our figure specifications.

# Author comment: Author response to Decision Letter 1

**DOI:** 10.1371/journal.pone.0358994.r002

4 Jun 2026

We sincerely thank you for the careful review of our manuscript and for the constructive comments and suggestions. We have carefully revised the manuscript to address all comments raised. The revisions have significantly improved the clarity, methodological transparency, and theoretical contribution of the paper.

Attachment Submitted filename: Response to Reviewers.docx

# Peer review: Decision Letter 1

**DOI:** 10.1371/journal.pone.0358994.r003

**Authors:** Gizachew Tiruneh

10 Jul 2026

Dear Dr. KAGIRI,

Thank you for submitting your manuscript to PLOS ONE. After careful consideration, we feel that it has merit but does not fully meet PLOS ONE’s publication criteria as it currently stands. Therefore, we invite you to submit a revised version of the manuscript that addresses the points raised during the review process.

Thank you for addressing the reviewers' comments thoroughly. I noticed several editorial issues, along with some more points that still need attention. Please see below.

Please submit your revised manuscript by Aug 24 2026 11:59PM. If you will need more time than this to complete your revisions, please reply to this message or contact the journal office at plosone@plos.org. When you're ready to submit your revision, log on to https://www.editorialmanager.com/pone/ and select the 'Submissions Needing Revision' folder to locate your manuscript file.

- A letter that responds to each point raised by the academic editor and reviewer(s). You should upload this letter as a separate file labeled 'Response to Reviewers'.
- A marked-up copy of your manuscript that highlights changes made to the original version. You should upload this as a separate file labeled 'Revised Manuscript with Track Changes'.
- An unmarked version of your revised paper without tracked changes. You should upload this as a separate file labeled 'Manuscript'.

If you would like to make changes to your financial disclosure, please include your updated statement in your cover letter. Guidelines for resubmitting your figure files are available below the reviewer comments at the end of this letter.

If applicable, we recommend that you deposit your laboratory protocols in protocols.io to enhance the reproducibility of your results. Protocols.io assigns your protocol its own identifier (DOI) so that it can be cited independently in the future. For instructions see: https://journals.plos.org/plosone/s/submission-guidelines#loc-laboratory-protocols. Additionally, PLOS ONE offers an option for publishing peer-reviewed Lab Protocol articles, which describe protocols hosted on protocols.io. Read more information on sharing protocols at https://plos.org/protocols?utm_medium=editorial-email&utm_source=authorletters&utm_campaign=protocols.

As the corresponding author, your ORCID iD is verified in the submission system and will appear in the published article. PLOS supports the use of ORCID, and we encourage all coauthors to register for an ORCID iD and use it as well. Please encourage your coauthors to verify their ORCID iD within the submission system before final acceptance, as unverified ORCID iDs will not appear in the published article. *Only*  the individual author can complete the verification step; PLOS staff *cannot*  verify ORCID iDs on behalf of authors.

We look forward to receiving your revised manuscript.

Kind regards,

Gizachew Tadele Tiruneh, Ph.D.

Academic Editor

PLOS One

Journal Requirements:

1. If the reviewer comments include a recommendation to cite specific previously published works, please review and evaluate these publications to determine whether they are relevant and should be cited. There is no requirement to cite these works unless the editor has indicated otherwise.

2. Please review your reference list to ensure that it is complete and correct. If you have cited papers that have been retracted, please include the rationale for doing so in the manuscript text, or remove these references and replace them with relevant current references. Any changes to the reference list should be mentioned in the rebuttal letter that accompanies your revised manuscript. If you need to cite a retracted article, indicate the article’s retracted status in the References list and also include a citation and full reference for the retraction notice.

Additional Editor Comments:

Dear authors,

Thank you for addressing the reviewers' comments thoroughly. I noticed several editorial issues, along with some minor points that may still need attention. Please see below

Abstract

1. Drop (UHC), as it appears only once in the Abstract

2. Similarly, spell out all abbreviations (e.g., Community Health Promoters [CHPs]) the first time they appear.

3. In the final sentence of the conclusion, remove the phrase "that cannot be generalised from Ethiopia's experience." The conclusion should focus on the study findings and implications rather than making comparisons with other settings.

Introduction

4. Consider restructuring the introduction to improve its logical flow: Paragraph 1: Present the problem statement by combining the current first and second paragraphs into a concise overview. Paragraph 2: Summarize what is currently known about the barriers and facilitators to eCHIS implementation, identify the knowledge gaps, and explain what remains unknown. Paragraph 3: Introduce the Kenyan Community Health Program and the eCHIS intervention. Paragraph 4: Present the rationale and significance of the study, followed by Paragraph 5 the study objectives.

5. Paragraph 3 is long. Try to trim or make 2 paragraphs

6. The fourth paragraph (Lines 65–71) largely repeats issues already presented in the second paragraph. Instead, dedicate this paragraph to clearly explaining the rationale, significance, and contribution of the study.

7. Several citations are not integrated clearly into the narrative: Line 12: The sentence beginning "[3] affirms that..." is unclear. Either mention the author's name or rewrite the sentence. The same issue occurs in: Line 18 ("For instance, a study by [4]..."); Line 23 ("...consistent with [5] findings..."); Line 66 ("A study by [1]..."); Line 130 ("...outlined by [13]."). Revise these sentences so that citations are incorporated appropriately.

8. Line 33: Provide the equivalent value in USD for KSh 2,500, indicating the exchange rate or base year used.

9. Ensure abbreviations are introduced only once: Line 43: Do not abbreviate Universal Health Coverage again if it has already been introduced. Line 79: Spell out Work System Framework (WSF) at first mention, and then use WSF consistently thereafter. Consequently, the full term does not need to be repeated in Lines 85 and 88.

Methods

10. Line 97, change “ECHIS” to “eCHIS”

11. Line 101, replace the word “was” with “were”

12. Line 102 provide additional description of the study settings

13. Line 108, replace (9–17) with 9-17

14. Line 109-110, don’t redefine Community Health Promoters(CHPs) and Community Health Assistants (CHAs). Please check these throughout the manuscript

15. The data collection period appears to be March 6, 2024, to April 10, 2024 (Line 119). Please revise the study period reported in Line 102 to ensure consistency.

16. Remove the sentences in Lines 112–113 describing IRB approval, as ethical approval is typically reported elsewhere. Instead, include details such as the language(s) used for data collection and where interviews were conducted (e.g., participants' homes or workplaces).

Findings

17. Begin the Results section with participants' characteristics before presenting the thematic findings.

18. Consider including a summary table that organizes the identified barriers and enablers according to each analytic domain. This would help readers quickly understand the main findings.

Discussion

19. Line 451: Do not redefine Work System Framework (WSF) if it has already been introduced earlier in the manuscript.

20. The third paragraph of the Discussion is lengthy. Consider splitting it into two paragraphs, with one dedicated specifically to the study's strengths and limitations.

21. Revisit the final paragraph. Avoid using the term "generalizable" in the conclusion. In addition, the paragraph states that the study provides four insights, but only three are actually presented. Please revise for consistency.

[Note: HTML markup is below. Please do not edit.]

Reviewers' comments:

Reviewer's Responses to Questions

**Comments to the Author**

Reviewer #2: All comments have been addressed

**********

2. Is the manuscript technically sound, and do the data support the conclusions?

Reviewer #2: (No Response)

**********

3. Has the statistical analysis been performed appropriately and rigorously?

Reviewer #2: (No Response)

**********

4. Have the authors made all data underlying the findings in their manuscript fully available?

The PLOS Data policy

Reviewer #2: (No Response)

**********

5. Is the manuscript presented in an intelligible fashion and written in standard English?

Reviewer #2: (No Response)

**********

Reviewer #2: (No Response)

**********

what does this mean?). If published, this will include your full peer review and any attached files.

If you choose “no”, your identity will remain anonymous but your review may still be made public.

**Do you want your identity to be public for this peer review?**  For information about this choice, including consent withdrawal, please see our Privacy Policy

Reviewer #2: No

**********

[NOTE: If reviewer comments were submitted as an attachment file, they will be attached to this email and accessible via the submission site. Please log into your account, locate the manuscript record, and check for the action link "View Attachments". If this link does not appear, there are no attachment files.]

To ensure your figures meet our technical requirements, please review our figure guidelines: https://journals.plos.org/plosone/s/figures

You may also use PLOS’s free figure tool, NAAS, to help you prepare publication quality figures: https://journals.plos.org/plosone/s/figures#loc-tools-for-figure-preparation.

NAAS will assess whether your figures meet our technical requirements by comparing each figure against our figure specifications.

# Author comment: Author response to Decision Letter 2

**DOI:** 10.1371/journal.pone.0358994.r004

7 Sep 2026

RE: SUBMISSION OF REVISED MANUSCRIPT

We sincerely thank you and the reviewers for the careful evaluation of our manuscript and for the constructive comments and recommendations provided. We appreciate the opportunity to revise the manuscript and believe that the revisions undertaken have substantially improved its clarity, methodological reporting, presentation of findings, and overall contribution.

We have carefully considered and addressed all the comments raised by the reviewers. A detailed point-by-point response has been prepared indicating how each comment was addressed and where the corresponding changes have been made in the revised manuscript.

Among the key revisions, we have strengthened the Introduction to provide clearer theoretical and empirical justification for examining sustained eCHIS usage and its socio-technical determinants. We have also clarified the application of the Work System Framework as the analytical lens guiding the study.

The Results section has been reorganised to begin with participants' characteristics before presenting the thematic findings. We have additionally included a summary table organising the principal enablers and barriers according to the four Work System Framework domains—participants, information, technology, and processes—to provide readers with a concise overview of the findings before the detailed thematic presentation.

The Discussion has also been strengthened by engaging more extensively with relevant eCHIS, digital health, and health information systems literature. The revised discussion more clearly distinguishes findings that are consistent with previous research, those that extend existing evidence, and the contribution of the Work System Framework in explaining how factors across participants, information, technology, and processes interact to shape consistent eCHIS usage.

Thank you for your time and consideration.

Attachment Submitted filename: Response to Reviewers.pdf

# Editor report: Decision Letter 2

**DOI:** 10.1371/journal.pone.0358994.r005

**Authors:** Gizachew Tiruneh

8 Sep 2026

Enablers and Barriers to Electronic Community Health Information System (eCHIS) Usage in Kenya: A Work System Framework Analysis of Frontline Health Worker Experiences.

PONE-D-25-49990R2

Dear Dr. KAGIRI,

We’re pleased to inform you that your manuscript has been judged scientifically suitable for publication and will be formally accepted for publication once it meets all outstanding technical requirements.

Within one week, you’ll receive an e-mail detailing the required amendments. When these have been addressed, you’ll receive a formal acceptance letter and your manuscript will be scheduled for publication.

An invoice will be generated when your article is formally accepted. Please note, if your institution has a publishing partnership with PLOS and your article meets the relevant criteria, all or part of your publication costs will be covered. Please make sure your user information is up-to-date by logging into Editorial Manager at Editorial Manager® and clicking the ‘Update My Information' link at the top of the page. For questions related to billing, please contact billing support.

If your institution or institutions have a press office, please notify them about your upcoming paper to help maximize its impact. If they’ll be preparing press materials, please inform our press team as soon as possible -- no later than 48 hours after receiving the formal acceptance. Your manuscript will remain under strict press embargo until 2 pm Eastern Time on the date of publication. For more information, please contact onepress@plos.org.

Kind regards,

Gizachew Tadele Tiruneh, Ph.D.

Academic Editor

PLOS One

Additional Editor Comments (optional):

Reviewers' comments:

# Editor report: Acceptance letter

**DOI:** 10.1371/journal.pone.0358994.r006

**Authors:** Gizachew Tiruneh

PONE-D-25-49990R2

PLOS One

Dear Dr. KAGIRI,

I'm pleased to inform you that your manuscript has been deemed suitable for publication in PLOS One. Congratulations! Your manuscript is now being handed over to our production team.

At this stage, our production department will prepare your paper for publication. This includes ensuring the following:

* All references, tables, and figures are properly cited

* All relevant supporting information is included in the manuscript submission,

* There are no issues that prevent the paper from being properly typeset

You will receive further instructions from the production team, including instructions on how to review your proof when it is ready. Please keep in mind that we are working through a large volume of accepted articles, so please give us a few days to review your paper and let you know the next and final steps.

Lastly, if your institution or institutions have a press office, please let them know about your upcoming paper now to help maximize its impact. If they'll be preparing press materials, please inform our press team within the next 48 hours. Your manuscript will remain under strict press embargo until 2 pm Eastern Time on the date of publication. For more information, please contact onepress@plos.org.

You will receive an invoice from PLOS for your publication fee after your manuscript has reached the completed accept phase. If you receive an email requesting payment before acceptance or for any other service, this may be a phishing scheme. Learn how to identify phishing emails and protect your accounts at https://explore.plos.org/phishing.

If we can help with anything else, please email us at customercare@plos.org.

Thank you for submitting your work to PLOS One and supporting open access.

Kind regards,

PLOS One Editorial Office Staff

on behalf of

Dr. Gizachew Tadele Tiruneh

Academic Editor

PLOS One
